# Can post-treatment oral cancer patients’ concerns reflect their cancer characteristics, HRQoL, psychological distress level and satisfaction with consultation?

**DOI:** 10.3332/ecancer.2020.1118

**Published:** 2020-10-08

**Authors:** Ainon Natrah Aminnudin, Jennifer Geraldine Doss, Siti Mazlipah Ismail, Ma Bee Chai, Marzuki Zainal Abidin, Cri Saiful Jordan Milano Basri, Nurshaline Pauline Kipli, Lee Chee Wei

**Affiliations:** 1 Community Oral Health and Clinical Prevention, Faculty of Dentistry, University of Malaya, 50603 Kuala Lumpur, Malaysia; 2 Oral Cancer Research and Coordinating Centre (OCRCC), University of Malaya, 50603 Kuala Lumpur, Malaysia; 3 Department of Oro-Maxillofacial Surgical & Medical Sciences, University of Malaya, 50603 Kuala Lumpur, Malaysia; 4 Oral Maxillo-Facial Surgery, Sultanah Aminah Hospital, 80100 Johore Bahru, Malaysia; 5 Oral Maxillo-Facial Surgery, Queen Elizabeth Hospital, 88200 Kota Kinabalu, Malaysia; 6 Oral Maxillo-Facial Surgery, Seberang Jaya Hospital, 13700 Penang, Malaysia; 7 Oral Pathology & Oral Medicine Unit, Sarawak General Hospital, 93586 Sarawak, Malaysia; 8 Oral Maxillo-Facial Surgery, Kuala Lumpur General Hospital, 50586 Kuala Lumpur, Malaysia; 9 Oral Health Program, Ministry of Health Malaysia (MOH), 62590 Putrajaya, Malaysia

**Keywords:** patient concerns inventory (PCI), health-related quality of life, psychological distress, post-treatment, oral cancer, web-based computerised, paper version, consultation

## Abstract

**Background:**

Oral cancer and its treatment impact patients’ post-treatment outcomes, challenging clinicians to manage them optimally. Addressing patients’ concerns is central to holistic patient-centred care.

**Objectives:**

This study aimed to determine post-treatment oral cancer patients’ concerns and its relationship with patients’ clinical characteristics, health-related quality of life (HRQoL), psychological distress and patient satisfaction with the follow-up consultation.

**Methods:**

A total of 85 oral cancer patients were recruited from a three-armed pragmatic RCT study on the patient concerns inventory for head and neck cancer (PCI-H&N), which was conducted at six hospital-based oral maxillofacial specialist clinics throughout Malaysia. Malaysians aged 18 years and above and on follow-ups from 1 month to 5 years or more were eligible. Patients completed the PCI-H&N, functional assessment of cancer therapy -H&N v4.0 and Distress Thermometer at pre-consultation and satisfaction questionnaire at post-consultation. The data were analysed descriptively; multiple linear regression and multivariate logistic regression analyses were used to determine possible predictors of patients’ HRQoL and psychological distress.

**Results:**

‘Recurrence or fear of cancer coming back’ (31.8%) was most frequently selected. 43.5% of patients selected ≥4 concerns. A significantly high number of concerns were associated with patients of ‘1-month to 1-year post-treatment’ (*n* = 84%; *p* = 0.001). A significant association existed between ‘time after treatment completed’ and patients’ concerns of ‘chewing/eating’, ‘mouth opening’, ‘swelling’, ‘weight’, ‘ability to perform’, ‘cancer treatment’ and ‘supplement/diet-related’. ‘Chewing/eating’ was predicted for low HRQoL (*p* < 0.0001) followed by ‘appearance’ and ‘ability to perform recreation activities’ (personal functions domain). Patients with high psychological distress levels were 14 times more likely to select ‘ability to perform recreation activities’ and seven times more likely to select ‘feeling depressed’. No significant association was identified between patients’ concerns and patients’ satisfaction with the consultation.

**Conclusion:**

Routine follow-up consultations should incorporate the PCI-H&N prompt list to enhance patient-centred care approach as the type and number of patients’ concerns are shown to reflect their HRQoL and psychological distress.

**TRIAL REGISTRATION**: NMRR-18-3624-45010 (IIR).

## Background

The life journey of oral cancer survivors is set by challenges before and after treatment. Although oral cancer is not amongst the global leading cancers as compared to other cancers (GLOBOCAN, 2018: lung cancer, female breast cancer, prostate cancer, colorectal cancer, stomach cancer and liver cancer) [1], the disfigurement and dysfunction of post-treatment of oral cancer affects the basic functional ability and causes problems with social functioning that interferes with patient’s daily lives [2–4]. As the tongue is the most commonly diagnosed and the mainstay of treatment [5], tongue resection results in impairment of speech, mastication, swallowing and breathing that significantly affect patients’ quality of life besides affecting their ability to communicate [4]. Since oral cancer is in the head and neck region, the impact on physical changes of post-treatment is easily noticed. This physical alteration of the patient’s face could lead to a life-long impact which specifically affects patients’ appearance, especially amongst female patients, which has a significant impact on their psychological well-being [6]. The impact on patients’ dentition not only affects patients aesthetically but also the main function of chewing. The inability to chew properly could lead patients to suffer malnutrition and having to be selective of their food choices [7].

Apart from the impact on the patients, cancer also affects the people around them [8]. The caregivers are mainly their spouses and family members, who often feel distress, especially amongst patients at an advanced cancer stage, as the caregivers’ personal daily function and social activities need to be adjusted to accommodate their caretaking duties for the patients [9]. Oral cancer patients at advanced cancer stage need more attention and support from family members as they are highly dependent on caregivers. Realising the importance of maintaining stable mental health to improve their quality of life, the supportive care approach has been integrated into patient management for holistic care for oral cancer patients and support for their caregivers [10].

Health-related quality of life (HRQoL) improvement in long-term cancer survivors undeniably requires health and service provision that caters to the individuals’ concerns. Current strategies are targeted to better improve the quality of life of oral cancer survivors, especially during the post-treatment follow-up care to increase positive outcomes and minimise knowledge gaps in cancer survivorship [11].

Oral cancer patients’ HRQoL outcomes are significantly associated with patient characteristics (race, gender and age), cancer characteristics (time since diagnosis, cancer stage and sites) and treatment-related characteristics (the extent of surgery, use of adjuvant chemotherapy or radiation therapy) [12–14]. In general, HRQoL deteriorates immediately after therapy and returns toward baseline by 1 year [13, 15]. Adverse HRQoL outcomes have also been associated with the existence of a feeding tube, tracheotomy and comorbid disease [13].

Furthermore, patients’ psychological health can also have a negative impact on their daily routines, reduce the HRQoL, increase suffering and decrease survival odds [16–18]. A higher level of psychological distress amongst post-treated oral cancer patients is associated with eating problems, fear of recurrence and fatigue, whereas, for younger patients with feeding tubes and having other comorbidities, staying alone and being off work caused higher levels of distress [19]. As such, the assessment of the psychological well-being of oral cancer patients and survivors during follow-up visits could render pertinent information for better clinical decision-making.

Providing the best quality health care has always been a great challenge for clinicians. As the majority of patients are detected at late cancer stages (stages III and IV), patient management is more complex [20, 5]. With the high patient volume in hospitals, clinicians are hard-pressed to achieve optimal outcomes from their communication with patients during limited consultation sessions [21, 22]. Often, this results in patients’ unmet needs not being addressed more so if patients are unwilling to voice their concerns due to various cultural and communication barriers [23–25].

In recent years, patient-reported outcome measures (PROMs) [26, 27] and question prompt lists (QPL) [28] have been of increasing interest to clinicians. The premise is that the patients’ experiences and concerns can be used as indicators of their overall well-being and coping ability with their disease and treatment received [29]. These instruments are intended to maximise overall patient outcomes at any phase of cancer management [26, 30] to evaluate patients’ satisfaction, improve patient-clinician communication and encourage shared decision-making during a consultation [31]. Unfortunately, most of the tools available to measure unmet needs do not cater to specific diseases which involve dental and nutritional needs. These specific needs are substantial amongst oral cancer patients as they are mostly affected due to the disease and treatment impact on their dental and eating functions. If these needs are neglected, it could lead to negative outcomes [27]. Currently, no other prompt list addresses dental needs except the Patient Concerns Inventory for head and neck cancer (PCI-H&N) [27, 32].

Rogers, El-Sheikha and Lowe [32] had initiated a PCI-H&N to be used during routine follow-up consultation. PCI-H&N is a prompt list of patient’s concerns that they wished to discuss with the clinicians, and it was first introduced in the form of computer-assisted technology using a touch screen (TST) version [32]. PCI-H&N can be employed as an item-specific PROM [26] and as a communication tool to encourage question asking, i.e., QPL [28]. The prompt list functions as an adjunct for more effective patient–clinician communication that acts as a guide during patient–clinician consultation, empowers shared decision-making, improves patient’s satisfaction and promotes multidisciplinary care with the premise to uncover patient’s unmet needs and improve patient outcomes [26, 29, 33]. However, environmental factors can restrict patient–clinician communication and lead to barriers in addressing patients’ unmet needs. The pandemic outbreak of coronavirus disease in 2019 (COVID-19) has created a need for new norms of patient approach as the usual face-to-face consultation between patient-clinician at clinics is currently being kept to a minimum. Guided by prompt lists, clinicians are able to discuss their patients’ concerns through structured telephone consultations [34, 35] as a suitable alternative to ensure the continuity of holistic patient care.

In Malaysia, PCI-H&N [30] had been translated and cross-culturally adapted in 2014 by Hatta *et al.* [34]. The present study used an adapted PCI-H&N, which is a combination of the latest version from Ghazali *et al* [31] and Hatta* et al.* [34]. The list of concerns is further described in the ‘Study instruments’ section. The present study utilised two versions, namely, paper and a computerised web-based version.

To date, assessing post-treatment oral cancer patients’ concerns, HRQoL and psychological distress is not routinely incorporated as a part of the follow-up consultation protocol in Malaysia. This exclusion is because most HRQoL and psychological questionnaires are time-consuming and tedious [37]. Incorporating the PCI-H&N in routine post-treatment follow-up consultation sessions in oral maxillofacial specialist (OMFS) clinics throughout Peninsular Malaysia, Sabah, and Sarawak could potentially help to address post-treatment oral cancer patients’ unmet needs. The inclusion will ensure a patient-centred management approach with enhanced patient–clinician communication, thus contributing to patients’ improved HRQoL and psychological distress levels.

This study aimed to determine whether post-treatment oral cancer patients’ concerns could reflect their cancer characteristics, HRQoL, psychological distress level and satisfaction with their consultations.

The hypothesis is that the number and type of patients’ concerns can significantly discriminate cancer characteristics, HRQoL, psychological distress level and satisfaction with their consultations.

## Materials and methods

Ethical approval was obtained from the Medical Research and Ethics Committee of the Ministry of Health Malaysia: NMRR-18-3624-45010 (IIR), and the Medical Ethics Committee, Dental Faculty. Permission to conduct the study in multiple OMFS hospital-based clinics in Peninsular Malaysia, Sabah, and Sarawak was obtained from the Oral Health Programme, Malaysia.

### Study design

**Figure 1.**
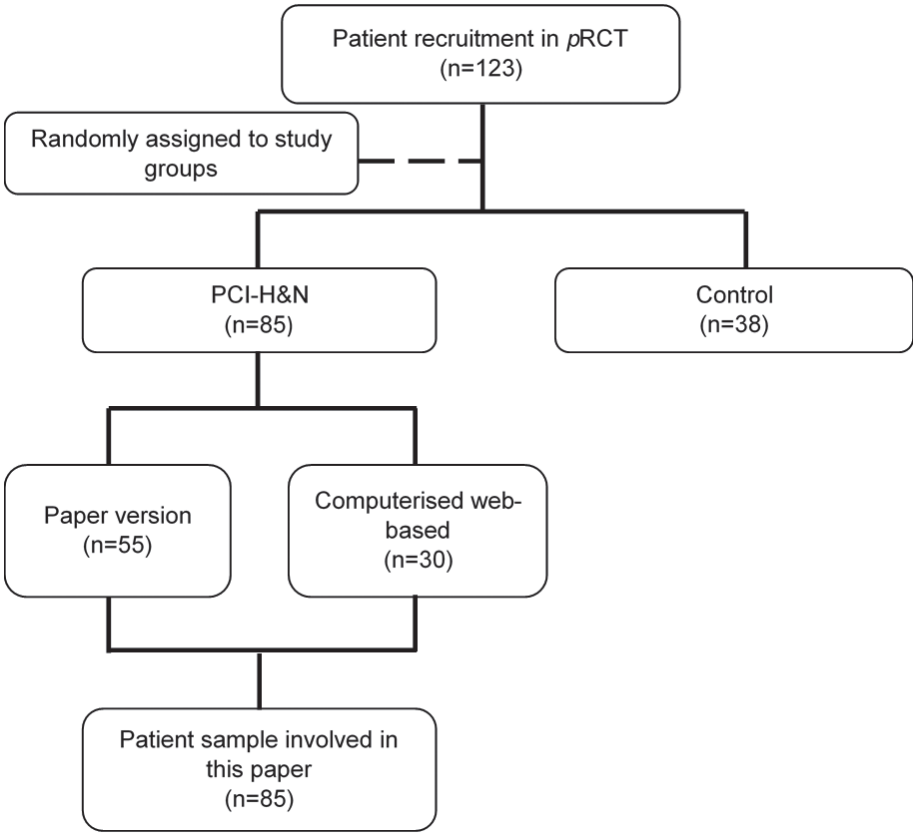
Post-treatment oral cancer patients involved in this paper (*n* = 85).

This study was a subset of a larger three-armed pragmatic randomised control trial (*p*RCT) assessing oral cancer patients’ concerns with post-treatment outcomes of health-related quality of life (HRQoL), psychological distress and satisfaction with a follow-up consultation. In this multicentre study, a total of 123 eligible post-treatment oral cancer patients were recruited, randomised and allocated to the three study groups of paper version (*n* = 55), computerised web-based version (*n* = 30) and control group (normal consultation practice) (*n* = 38). The content of PCI-H&N is the same regardless of their two different versions (paper version and computerised web-based version). These two groups were combined for a more meaningful data interpretation in assessing the post-treatment oral cancer outcomes amongst the PCI-H&N and non-PCI-H&N used. The present study focuses only on the PCI-H&N group which addressed post-treatment oral cancer patients’ concerns to determine the association of patients’ concerns with the patients’ clinical characteristics, HRQoL, psychological distress and satisfaction with their follow-up consultations. The study flow of patients involved in this study is shown in Figure 1.

All eligible post-treatment oral cancer patients present for their follow-up appointments from April to December 2019 were recruited in this study, and written informed consents were obtained from all enrolled patients. A pilot test and training session for participating clinicians and assistants were conducted before this study.

Patients were given a set of questionnaires consisting of the patients’ profile, functional assessment of cancer therapy scale (FACT-H&N v4.0) and Distress Thermometer (DT) at pre-consultations. In addition, a satisfaction questionnaire was given to patients after they had completed their consultations with the clinicians.

### Study sample

The target study population was follow-up post-treatment oral cancer patients from six identified hospital-based OMFS clinics (five government hospitals and one institutional hospital). These study sites were selected as they are the leading tertiary referral centres for oral cancer in Malaysia. Eligible patients were (i) Malaysians aged 18-year old and above, (ii) post-treatment oral cancer patients who had completed their oral cancer treatment (surgical/radiotherapy/chemotherapy or combination) at least 1 month earlier and (iii) those under routine follow-up in the identified hospitals. Patients were excluded if they (i) had known psychiatric or mental condition, (ii) had a recurrence of oral cancer during the data collection period and (iii) were unaccompanied and had significant difficulty in hearing, reading or speaking.

### Study instruments

Three patient self-administered questionnaires (with assistance when needed) and a PCI-H&N were used in this study besides the questionnaire of patients’ profiles (gender, age, ethnicity and religion, marital status and education level, cancer sites, staging, types of treatment and time after treatment completed). The four instruments used in this study are further described in the following sections.

(1) Patient concerns inventory head and neck (PCI-H&N)

This questionnaire consists of 52 patient concerns grouped into seven domains (i) physical status (23 items), (ii) personal functions (six items), (iii) treatment-related (six items), (iv) social care and social well-being (seven items), (v) economic status (one item), (vi) emotional status (eight items) and (vii) spiritual well-being (one item). It was adapted from Hatta *et al*’s list of 43 items [36] (cross-culturally adapted to Malaysia) with an additional nine items from Ghazali *et al* [33] (i.e., ‘breathing’, ‘coughing’, ‘carers’, ‘dependant/children’, ‘home care/district nurse’, ‘coping’, ‘self-esteem’, ‘fear of adverse events’ and ‘mood’).

The final prompt list was face and content validated (by a public health specialist and an OMFS specialist) and translated into the Malay language using the forward- and back-translation method. This instrument was administered into two versions, either the paper version or the computerised web-based version using a touch screen tablet.

(2) Functional assessment of cancer therapy scale (FACT-H&N v4.0) Questionnaire

A validated and cross culturally adapted Functional Assessment of Cancer Therapy Scale (FACT-H&N v4.0) [38] was used to measure patients’ health-related quality of life. FACT-H&N v4.0 consists of six domains with 49 self-reported questions using the 5-point Likert scale (not true at all, somewhat true, quite true, true and very true). The domain categories are (i) physical condition (PWB), (ii) social/family relationship (SWB), (iii) emotional well-being (EWB), (iv) personal functions (FWB), (v) head and neck subscale questions and (vi) Malaysian-added questions (MAQ). All the questions are related to patients’ experience for the past 7 days. A general question on patients’ self-rated overall HRQoL and a transition judgement question (on patients’ self-rated change in HRQoL of the current visit compared to the previous visit) was incorporated at the end of this questionnaire. Scoring was based on the FACT scoring guideline [38]. In this study, the question on sexual relationships (GS7) was excluded due to meagre response, and the question on betel quid chewing habit (MQ6) from the additional set of Malaysian-added questions was also excluded as it is related to risk habit practice. The total HRQoL score used for data analysis was the FACT-H&N-MAQ total score.

(3) DT questionnaire

DT is a single-item instrument to measure distress and depression experienced amongst patients for the past 7 days and had been culturally adapted and validated for the Malaysian population [39]. DT consists of two sections, i.e., a visual analogue scale and a checklist of problems experienced in the past 7 days. This study adopted a cut-off point of 4 as suggested by Yong *et al.* [38] and Ghazali *et al.* [40]. Groups were categorised as ‘low risk’ (score 0–3) and ‘at risk’ (score 4–10).

(4) Patients’ satisfaction questionnaire

A set of questions regarding the patient’s satisfaction with the quality of the consultation session was adapted from the literature review and previously validated questionnaires [41–44]. The final questionnaire consisted of seven self-rated statements with a 5-point Likert scale response option of very satisfied, satisfied, neither, dissatisfied and very dissatisfied and two open-ended questions. This questionnaire was face and content validated and forward- and back-translated into the Malay language, and its reliability was assessed during the pilot test (Cronbach’s *α* = 0.83).

### Data analysis

The analysis was carried out using the IBM statistical package for the social sciences (SPSS) 20.0, and the statistical significance level was established at *p* < 0.05. Pearson Chi-square, Fisher’s exact test, Mann–Whitney test and an independent sample *T*-test were used accordingly to analyse the patients’ profile and the association between PCI-H&N (by scores and by domains) and the (i) patients’ profile, (ii) HRQoL, (iii) psychological distress and (iv) patients’ satisfaction with their follow-up consultations. Multilinear regression and multivariate logistic regression analyses were performed to determine possible predictive concerns associated with patients’ HRQoL and psychological distress levels.

## Results

A total of 102 post-treatment oral cancer patients have presented for follow-up appointments at the six OMFS clinics. Of these, 17 patients did not agree to participate in this study rendering a response rate of 83% (*n* = 85/102).

Eighty-five post-treatment oral cancer patients were recruited on their follow-up appointment days into the two study groups who used PCI-H&N (paper version and computerised web-based version). Females (61.2 %) and Malay (42.4%) were predominant, and the mean (SD) age for patients in this study was 58.9 (SD = 12.8) years. The most-reported sites of the primary cancer were tongue (*n* = 49) followed by buccal mucosa (*n* = 12) and lips (*n* = 7). Patients were mostly diagnosed at the early stage of cancer, with 37.6% in stage 1 and 29.4% in stage 2. More than half (52.9%) had both surgery and adjunct treatment (radiotherapy with/without chemotherapy), and almost 46% had surgery only. Most patients in this study were at their post-treatment follow-up from more than 1 year until <3 years (41.8%) and more than 1 month–1 year (28.4%), with the overall sample range of 1 month until 15 years after completing treatment.

In this study, patients’ concerns from both PCI-H&N versions were grouped for more meaningful data interpretation. The physical status domain (*n* = 59) was highly selected amongst post-treatment oral cancer patients, followed by emotional status (*n* = 36), cancer treatment-related (*n* = 23), personal functions (*n* = 22), social care and social well-being (*n* = 7), economic status (*n* = 6) and spiritual well-being (*n* = 1).

Most frequently selected by the patients (Figure 3) in descending order were ‘recurrence or fear of cancer coming back’ (31.8%), issues of their ‘dental problems’ (24.7%), ‘chewing’ (21.2%), ‘dry mouth’ (21.2%) and ‘sore mouth’ (16.5%). On the contrary, the least selected concerns were ‘mood swings’, ‘spiritual/religious aspects’, ‘relationships issues’, ‘carers′ aspects’ and ‘vomiting/sickness’ (1.2%, respectively). Items not selected at all by any of the patients included ‘PEG tube’, ‘sexuality/intimacy’ and ‘lifestyle’. The median (IQR) number of the PCI-H&N items selected was three (1–5.5), ranging from zero to 17 items. This study showed that 23.5% of the patients did not select any items, 33% selected one to three items, and 43.5% selected four or more concerns.

The Pearson Chi-square test and independent sample *T*-test were both used to analyse the association between the number of patient’s concerns with the patient’s sociodemographic background. However, there were no significant findings.

‘Time after treatment completed’ (patient cancer characteristic) was significantly associated (*p* < 0.001) with the number of concerns selected. This study suggested that a high number of concerns were associated with the earlier phase of post-treatment because a significant 84% amongst the 1-month until 1-year post-treatment patients had a high number of concerns selected, whereas fewer issues of concerns (0–3 concerns) were selected by 75% of those who had completed treatment 3–5 years ago.

Three of the seven domains were significantly related with the ‘time after treatment completed’ as shown in Table 1 [physical status (*p* < 0.001), personal functions (*p* < 0.05) and treatment-related (*p* < 0.001) domains]. These patients’ specific concerns, i.e., ‘chewing/eating’, ‘mouth opening’, ‘swelling’, ‘weight’, ‘ability to perform daily routines’, ‘cancer treatment’ and ‘health supplement/diet-related issues’ were significantly associated with ‘time after treatment completed’. The analysis as shown in Table 1 also suggested that the significant PCI-H&N specific concerns were highly significant amongst patients in the category of ‘1 month until less than 1 year’ after completing treatment (Fisher’s exact test, *z*-score > 1.96^). This group of patients had raised concerns on ‘chewing/eating’ (*n* = 10), ‘recurrence/fear of cancer coming back’ (*n* = 9), whereas seven had selected ‘dental health/teeth’, ‘dry mouth’, ‘cancer treatment-related’ and ‘mouth opening’ concerns.

**Figure 2.**
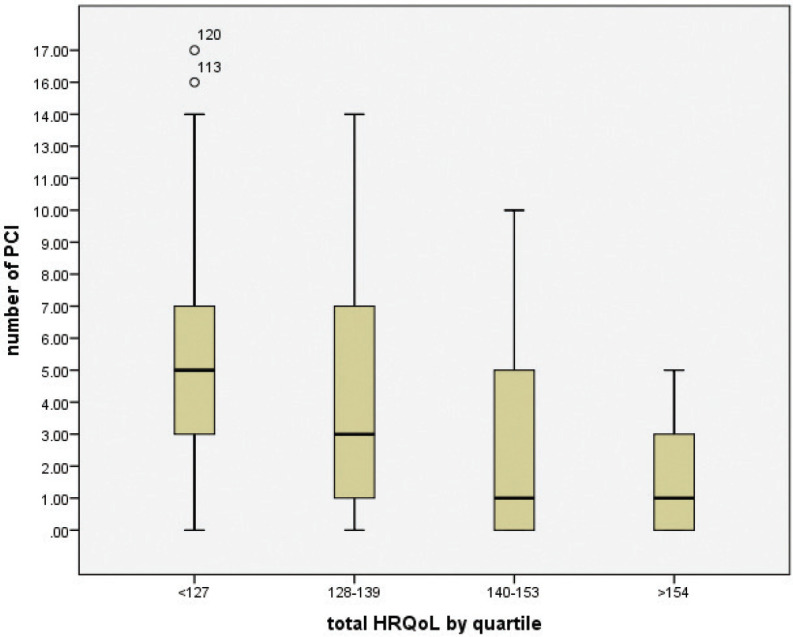
Boxplot of a numbrs of patients’ concern by HRQoL quartile (*n* = 85).

**Figure 3.**
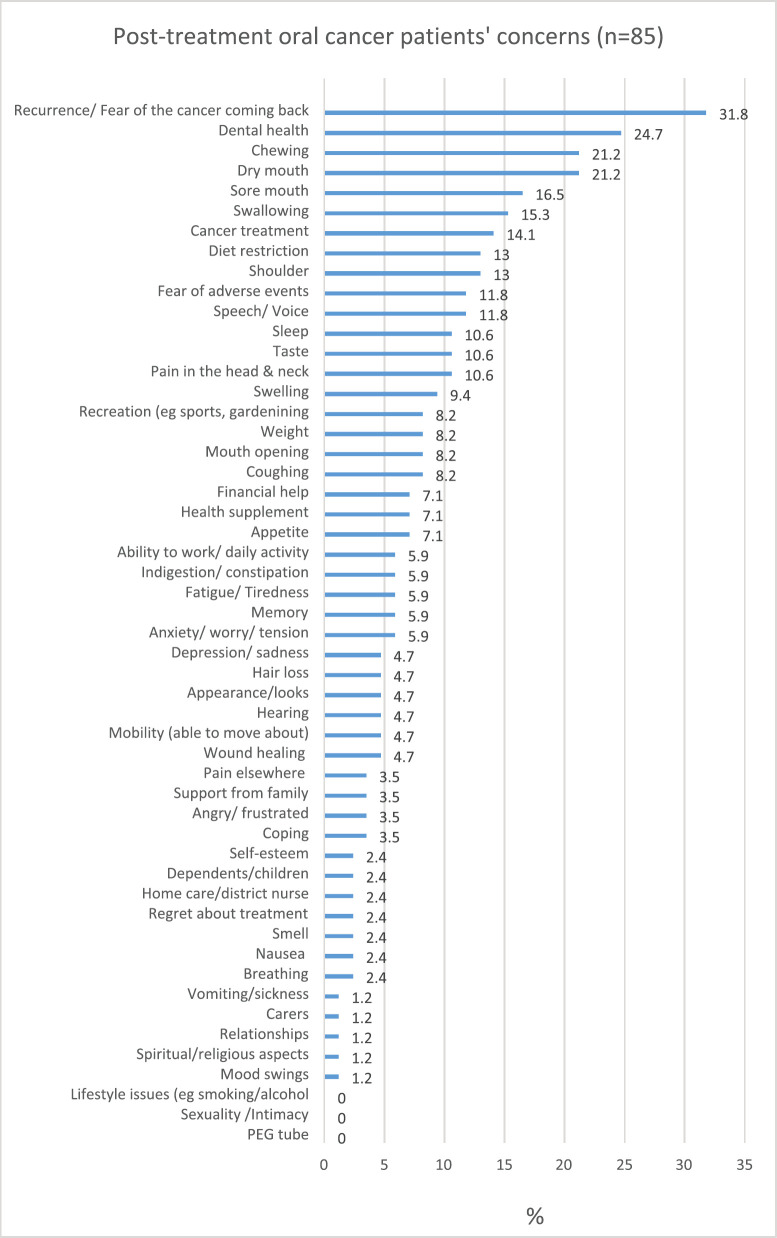
Post-treatment oral cancer patients’ concerns.

Approximately 70% of post-treatment oral cancer patients with lower HRQoL scores (77–140) had significantly more numbers of concerns (4–17 items) and vice versa (Table 2) (*p*-value = 0.003 and Spearman’s correlation *r* = 0.466). This study found that patients raised more concerns when they had low HRQoL scores. Figure 2 shows that a high number of patients’ concerns with median (IQR) of 5 (3–7) (*n* = 23) were reported amongst those with median (IQR) HRQoL of 113 (91–125).

In terms of domain, ‘patients’ personal status’ and ‘physical functions’ were significantly related. Personal functions strongly predicted patients’ lower HRQoL (standardised coefficients = −0.601, *p* < 0.0001). A significant 38% variance in HRQoL score was explained by patients’ personal status and physical functions domains (multiple linear regression: *p* < 0.0001; *R*^2^ = 0.393, adjusted *R*^2^ = 0.338, *F* (7, 77) = 7.133).

However, multiple linear regression analysis on PCI-H&N specific items revealed that a significant 54% variance in patients’ total HRQoL score was explained by ‘chewing and eating’, ‘appearance’ and ‘ability to perform recreation activities’ (*p* < 0.0001; *R*^2^ = 0.542, adjusted *R*^2^ = 0.480, *F*(10,74) = 8.77). This analysis suggested that patients’ HRQoL can be moderately predicted by ‘appearance’ and ‘ability to perform recreation activities’, with ‘chewing and eating’ as a stronger predictor for low HRQoL score (standardised coefficients = −0.395, *p* < 0.0001), as shown in Table 3.

At a cut-off point of four concerns, psychological distress level was not significantly associated with the number of patients’ concerns (*p*-value > 0.05). This study demonstrated a higher proportion of patients with low-risk psychological distress levels regardless of the number of concerns selected.

Oral cancer patients’ emotional status was the only predictor in the PCI-H&N domains that were significantly related to their DT level. This study suggested that the probability of patients with a higher tendency of psychological distress (DT > 4) was 24% more if they had emotional concerns. Other PCI-H&N domains did not demonstrate any significant association with DT levels.

In terms of specific concerns selected, patients who selected ‘ability to perform recreation activities’ and ‘feeling depressed/sad’ (multivariate logistic regression: seven and 14 times respectively) were more likely at the risk of psychological distress than patients who selected other PCI-H&N items (Table 4). Statistical assumptions of multicollinearity had suggested that multicollinearity was a possible issue with *p* < 0.05 and Pearson’s *r* < 0.80.

No significant difference was observed between patients’ satisfaction with their follow-up consultations and the number of patients’ concerns selected. Nevertheless, 53% of satisfied post-treatment patients had a fewer number of concerns (0–3 items). This study showed no significant association between the PCI-H&N domains and post-treatment oral cancer patients’ satisfaction with their follow-up consultations.

## Discussion

To date, this is the first study conducted to determine post-treatment oral cancer patients’ concerns and its relationship with patients’ clinical characteristics, HRQoL, psychological distress and patient satisfaction during their follow-ups. The strengths and limitations of this study are discussed in the following sections.

This study was conducted at multiple study sites to represent patients’ variation in terms of ethnicity and practices in Malaysia [45, 46]. Thus, the outcomes interpreted in this study are deemed to reflect the post-treatment oral cancer population throughout the country. Furthermore, the sample was drawn from a *p*RCT study of PCI-H&N, which facilitated data collection under real circumstances at the selected OMFS clinics. Patients’ concerns derived from the two PCI-H&N versions were combined for a more meaningful data interpretation as the content and function of both versions are the same. This step is supported by a previous study [47] and from the main unpublished *p*RCT PCI-H&N study in Malaysia.

Insights into this study’s findings may be of added value to clinicians for improving their management of their post-treatment oral cancer patients. However, this study’s outcomes should be interpreted within the study limitations.

The findings are limited by the small sample size, which is reflective of the low oral cancer incidence in Malaysia [48]. Other similar studies also reported small sample sizes [36, 49–51]. In a period of 8 months, only 85 eligible patients agreed to participate in the present study. Moreover, the number of patients recruited in this study was dependent on those who presented for their follow-up consultations. The majority of the non-presented patients were older age groups who depended on their carers or children to bring them to the clinics and others who chose to continue their follow-up appointments at their primary hospitals instead of the tertiary centres for logistic reasons.

Data were also obtained through self-reported questionnaires, which was another limitation in this study. A self-reported questionnaire is highly dependent on the participants’ honesty in answering the questions [52]. Self-reported data may have resulted in some under-reporting. Proxies assisted a few patients in completing the questionnaires that may have led to information bias.

The median number of three concerns selected by patients in the present study was lower than the latest PCI variation study by Rogers *et al* [53], which reported with a median (IQR) of 5 (2–10) concerns (range of 0 to 48 items) [53]. In addition, a lower proportion of patients in the present study selected six or more concerns compared to nearly half in the latter study [53]. These findings may imply that Malaysian post-treatment oral cancer patients had better post-treatment coping mechanism with a lower impact on their daily routines. Nevertheless, it could also be reflective of the conservative customs of upbringing that do not encourage sharing the worries with others, especially outsiders.

Patients’ concerns were mostly regarding their physical status, treatment-related issues and emotional domains, specifically on ‘fear of cancer coming back’, ‘dental health’, ‘chewing’, ‘dry mouth’, ‘sore mouth’, ‘swallowing’, ‘cancer treatment’, ‘diet restriction’, ‘shoulder’, ‘speech/voice’ or ‘fear of adverse events’. These concerns concurred with other studies [8, 53, 54]. Although differences in the ranking order of concerns were noted in comparison with recent findings by Rogers *et al* [53], dental health and oral functions constituted the six most frequent patient concerns, very much related to the early phase of post-treatment. The multidisciplinary dental team mostly manages these concerns.

Understandably, ‘fear of cancer coming back’ was noted as the most frequent concern amongst more than one-third of patients in both studies (Table 5). This finding implies that post-treatment oral cancer patients across different countries, regardless of ethnicity and religion, are potentially at risk of mental health issues such as ‘fear of recurrence’, ‘distress’, ‘anxiety’ and ‘depression’ [55]. ‘Fear of cancer recurrence’ is acknowledged as one of the symptoms of distress [56]. Based on a qualitative study amongst post-operative head and neck cancer patients, feelings of fear and anxiety were mostly due to uncertainty of the future, death, costs of treatment and operative procedures when they heard about their diagnosis [57]. However, it contrasts with the finding of a previous local study which reported that most frequent concerns of post-treatment oral cancer patients were of physical status (94.4%), specifically chewing (48.6%) [36].

Issues such as sexuality/intimacy or lifestyles habits (smoking/alcohol) were not highlighted at all by patients presumably because these issues are more private topics, in which, perhaps, patients felt was inappropriate to discuss with their clinicians [32, 58]. Moreover, most clinicians tend not to address intimacy issues with patients [59, 60]. Although PCI-H&N is not a diagnostic tool, this prompt list could assist the clinicians to appreciate their patients’ concerns or worries and further manage it with their best possible capacity [40].

This study revealed a significant association between the number of PCI-H&N items selected and the ‘time after treatment completed’ (*p* < 0.001). This association is evident because oral cancer patients experience challenges from the time of diagnosis, through treatment, after treatment and throughout their remaining life journey [61]. This study observed that a significantly high number of concerns were strongly associated with patients of ‘1-month to 1-year post-treatment’. It concurs with a study conducted by Shunmugasundaram *et al* [27], whereby patients’ unmet needs are influenced by time from treatment completed amongst those in the immediate post-treatment phase as opposed to long-term survivorship. Higher unmet needs suggest that patients have more concerns that they wish to discuss during the follow-up consultation. On the other hand, fewer concerns were demonstrated amongst those who had completed treatment between 3 and 5 years. This difference could probably be that they had learned to adapt to their new situation by accepting and coping with the challenges to maintain social interaction [62], and some survivors (*n* = 71%) managed to resume their life as before treatment by returning to work within 6 months’ post-treatment [63].

‘Time after treatment completed’ was also significantly associated with PCI-H&N domains, mainly physical status, personal functions and treatment-related domains. These three domains were highly associated with patients at ‘1 month till less than 1-year post-treatment’. Those in the early recovering phase of post-treatment had mostly ‘dental related issues’ (eating, mouth opening), ‘swelling’, ‘weight’, ‘ability to perform daily activities’, ‘cancer treatment-related issues’ and ‘health supplement or dietary’ concerns. In another study, the common issues at the early stages were ‘fear of recurrence’, ‘dental health/teeth’, ‘taste’, ‘salivation’, ‘chewing’, ‘swallowing’, ‘mouth opening’, ‘fatigue’, ‘sleeping’, ‘speech’ and ‘pain’ [8, 54]. ‘Time after treatment completed’ was observed to be significantly related to cancer treatment-related issues (cancer treatment, regret about treatment, PEG tube, wound healing, health supplements and diet restriction). This finding is not uncommon as post-treatment oral cancer patients would have more issues about the type of treatment received. This finding concurred with the qualitative longitudinal study that reported that post-treatment patients had mostly nutritional concerns [64]. Almost 42% of post-treatment patients (of 5 years and more) in the present study had more than four concerns, possibly due to the cumulative effect and permanent disability related to their disease and the treatment received [65, 66]. As such, from the point of diagnosis and throughout cancer treatment and post-treatment, using a prompt list, for example, the Patient Concerns Inventory that assists cancer patients in identifying their concerns and seeking relevant information during the consultation session would be beneficial [67].

Patients’ HRQoL deteriorated at immediate post-treatment and subsequently improved slowly towards baseline scores after 1 year [68]. This finding explained the decreasing trends of patients’ concerns as they progressed in their recovery. The largest PCI study from different OMFS follow-up clinics around the world reported that a number of PCI items were strongly associated with the overall patients’ QoL, whereby 25% of patients who had selected five to nine items have less than good overall QoL [53].

Patients’ physical domain and personal functions were significantly associated with their HRQoL. In a different study, PCI-H&N concerns were found to be reflective of patient-centred issues with a statistically significant association with social and physical domains [51]. Since validated HRQoL domains guided the development of this prompt list, the issues selected by patients can inevitably act as clues for clinicians to gauge their patient’s HRQoL and health status without the use of a validated HRQoL questionnaire which tends to be burdensome, time-consuming and requiring extra resources [37].

Amongst the physical status and personal functions domains, ‘chewing/eating’ was the most substantial concern that predicted patients’ HRQoL. Given this strong association and similar findings of the impact of ‘chewing/eating’ on HRQOL in another study [48], this dental concern warrants special attention of clinicians and highlights the importance of multidisciplinary dental teams in its management.

Patients’ concerns, namely ‘appearance’ and ‘ability to perform recreation activities’, were also significant predictors of post-treatment oral cancer patients’ HRQoL. This result is not surprising as the majority of patients in the present study were of the younger age group who often prioritised aesthetics and fitness. Moreover, physical disfigurement caused by oral cancer and its treatment sometimes leads to life-long impacts, notably affecting patients’ appearance. In another study [64], ‘appearance’ was highly reported amongst female patients and had a significant impact on their psychological well-being. Likewise, in the present study, females, although few in numbers, also highlighted concerns of ‘appearance’.

The present study also suggested that if patients selected more than four concerns, it could indicate to the clinicians that the patients had a lower HRQoL. Improvement in managing oral cancer survivors can be achieved by continuously monitoring patients’ concerns at each follow-up visit to cater to patients’ individualised needs [69, 6]. However, more importantly, as a clinician, it can provide a better understanding of the impact of oral cancer treatment towards patients’ quality of life [70].

This study suggested that ‘post-treatment patients’ cancer staging’ and ‘time since completion of treatment’ were associated with the risk of psychological distress although the number of concerns was not associated. Psychological distress was also found to be significantly associated with the ‘emotional status’ domain, which was predicted by one specific item. Although the patients in the present study mostly reported on ‘recurrence or fear of cancer coming back’ (emotional domain), only ‘feeling depressed’ was significantly associated with their psychological well-being. This finding concurred with another study that reported significance with the emotional domain, too [53]. Mental issues such as fear of recurrence, distress, anxiety and depression occur more frequently amongst post-treatment oral cancer patients and can be prolonged as an impact from the disease and its treatment [55].

Nevertheless, another study had shown a significant association between psychological distress and ‘physical and functional well-being’ domain [40]. Similarly, in the present study, patients’ functional well-being was also significantly associated with psychological distress although only predicted by one specific item, namely ‘ability to perform daily activities’. This outcome could reflect the differences in cultural and social norms across western and eastern populations. Other studies had reported a significant association between the patients’ psychological distress and other specific items of personal functions domain: ‘sleep’ [40], ‘appearance or disfigurement’ [66] and ‘fear of recurrence, and interference with activities’ [71]. Furthermore, unlike in the present study, western patient populations were more likely to express a higher number of concerns, which was significantly associated with a risk of having psychological distress than eastern patients with a lesser number of concerns [40].

In clinical practice in Malaysia, a DT level of four and above suggests that a patient has signs of distress, thus requiring further referral for psychiatric management [39]. DT questionnaire used in an earlier study revealed that the most frequent issues contributing to the risk of psychological distress were related to ‘financial aspect’, ‘worry’, ‘nervousness’, ‘getting around’ and ‘sleep’, whereas those who were at risk for high distress were more likely to endorse problems related to worry [72]. Given the significant association found between the emotional domain and specific items (‘ability to perform daily activities’ and ‘feeling depressed’) in the present study, the PCI-H&N could be used as an indicator by clinicians to detect the early signs of significant psychological distress problems with a cut-off point of more than four items [40]. Nonetheless, the present study observed a non-significant association between the number of concerns and psychological distress, possibly due to the small sample size, thus warranting this to be further explored in future studies. The potential of PCI-H&N to act as a screening tool should also be explored in the future, given these outcomes. Psychological issues amongst post-treatment oral cancer patients are undoubtedly relevant and universal, requiring further psychological support services [73]. Up until now, it is not a routine practice to assess an oral cancer patient’s psychological distress level by using questionnaires on psychological health status either at the diagnosis phase or during routine post-treatment consultation. Since this study and other similar studies [40, 74] have shown that post-treatment oral cancer patients can have mental health impacts, therefore, clinicians should anticipate these significant psychological distress problems before initiating oral cancer treatment. The importance of supportive care, for example, reassurance, cannot be overemphasised and may enable patients to cope with the impacts of their treatment more effectively.

Various barriers exist in establishing good patient–clinician communication, especially during follow-up consultations, which indirectly leads to patients’ unmet needs not addressed, under-reporting their concerns and possibly their reluctance to share their worries [24, 25, 74, 75]. Patients were satisfied more when their expectations and concerns are addressed [76]. This factor shifts the emphasis on health providers’ ability to respond to their patients’ needs and preferences [77].

Most of the patients in the present study were satisfied with their follow-up consultation sessions. The fact that these satisfied patients selected fewer concerns (0-3 items) and most clinicians involved in this study were the patients’ regular clinicians during their routine follow-ups could be possible reasons for this finding. Thus, it can be inferred that both patients and clinicians had developed a good rapport which meets the patients’ needs [76]. Although not significant, it was observed that the patients were satisfied with the consultation discussions regarding their physical status and emotional domains.

## Conclusions

PCI-H&N is a prompt list that has the potential to be integrated routinely into clinical practice during post-treatment head and neck consultation in hospital-based OMFS clinics. Besides providing clinicians with an overview of their patients’ concerns, the list could also predict their patients’ health-related quality of life and psychological distress levels as a part of their oral cancer post-treatment outcomes. It seems plausible then that the success of the oral cancer treatment could be measured not only by clinical outcomes alone but also on its impact on patients’ health-related quality of life, including their psychological well-being. The present study has proven, within limitations, that the simple prompt list of PCI-H&N can be used as an individualised approach to assessing patients’ health-related quality of life and psychological distress in oral cancer patient post-treatment management. The variation in how PCI can be conducted to overcome any possible barriers has proven its multifunction purposes. Providing the best holistic patient-centred care will not be compromised as the prompt list can be tailored to current needs.

Thus, there is a potential future benefit in utilising this prompt list during post-treatment oral cancer follow-up consultations. The present study had proven, within limitations, that the simple prompt list of PCI-H&N can be used as an individualised approach to assessing patients’ health-related quality of life and psychological distress in oral cancer patient post-treatment management.

## Conflicts of interest

The authors declare that they have no conflicts of interest.

## Funding

Self-sponsored.

## Figures and Tables

**Table 1.** Significant association between cancer characteristic (‘time after treatment completed’) and PCI-H&N items selected (*n* = 85).

‘Time after treatment completed’	Physical status domain (*p* < 0.001)				Personal function domain (*p* < 0.05)	Treatment-related domain (*p* < 0.001)	
	Chewing/Eating (*n*;% )	Mouth opening (*n*;% )	Swelling (*n*;% )	Weight (*n*;% )	Ability to perform (*n*;% )	Cancer treatment (*n*;% )	Supplement/ diet related (*n*;% )
1 month till less than 1 year	10 (11.8)^	6 (7.1)^	5 (5.9)^	4 (4.7)^	5 (5.9)^	7 (8.2)^	4 (4.7)^
1 year till less than 3 years	3 (3.5)	0	0	0	0	2 (2.4)	0
3years till less than 5 years	0	1 (1.2)	0	1 (1.2)	0	0	0
5 years and more	2 (2.4)	0	1 (1.2)	0	0	0	0
*p*-value	0.003	0.002	0.013	0.021	0.005	0.015	0.015

Statistical analysis: Fisher’s exact test, *z*-score > 1.96^.

**Table 2.** Numbers of patients’ concerns based on their HRQoL, psychological distress and satisfaction with consultation.

Numbers of PCI selected	Total HRQoL		Psychological distress		Satisfaction with consultation	
	Low (77–140) *n* (%)	High (141–167) *n* (%)	DT level <4 *n* (%)	DT level > 4 *n* (%)	Very satisfied/satisfied *n* (%)	Neither/dissatisfied/very dissatisfied *n* (%)
Low number (0–3)	18 (37.5)	30 (62.5)	40 (83.3)	8 (16.7)	45 (32.9)	3 (3.5)
High number (4–17)	26 (70.3)^	11 (29.7)	27 (73.0)	10 (27.0)	34 (40.0)	3 (3.5)
*p*-value	0.003*		0.246		0.740	

Statistical test: Pearson’s chi-square; *p*-value<0.05*; *z*-score > 1.96^

**Table 3.** Multiple linear regression analysis of PCI-H&N items associated with patients HRQoL (*n* = 85).

Items	B	95% CI	*β*	*sr*^2^	*p*-value
Constant	147.42				
Physical status					
Chewing/Eating	−20.54	−31.00, −10.07	−0.395	0.095	0.000
Personal function					
Appearance	−28.93	−45.99, −11.88	−0.289	0.071	0.030
Ability to perform recreation activities	−16.80	−31.09, −2.50	−0.218	0.034	0.022

Statistical test: Multiple linear regression; *p* < 0.05

**Table 4.** Multivariate logistic regression analysis of PCI-H&N items associated with patients’ psychological distress level (*n* = 85).

PCI-H&N items	Crude OR	Adjusted OR	95% CI	*p*-value
Constant	−2.21	0.11		
Personal function				
Ability to perform recreation activities	1.95	7.03	1.161, 42.606	0.034
Emotional status				
Feeling depress/ sadness	2.66	14.34	1.152, 178.51	0.038

Statistical test: Multivariate logistic regression; *p* < 0.05

**Table 5.** Top 10 concerns by ranking between two studies.

Aminnudin *et al*, 2020	%	Rogers *et al*, 2019	%
Fear of cancer coming back	31	Fear of cancer coming back	39
Dental health	25	Dry mouth	37
Chewing/ eating	21	Chewing/Eating	29
Dry mouth	21	Swallowing	26
Sore mouth	16	Speech/Voice	25
Swallowing	15	Dental health	25
Cancer treatment related	14	Fatigue/ tiredness	23
Diet restriction	12	Salivation	22
Shoulder	12	Pain in head and neck	20
Speech/Voice	11	Cancer treatment related	20
